# The External Use of Chloroform in Labor

**Published:** 1885-01

**Authors:** 


### THE EXTERNAL USE OF CHLOROFORM IN LABOR.

An article, entitled "External Use of Chloroform in Labor," by Dr. A. Svanberg, Sweden, translated by Dr. C. W. Johnson, Chicago, appeared in the columns of "Selections," December number, 1884, CHICAGO MEDICAL JOURNAL AND EXAMINER.

As at present informed, modern obstetrical literature contains nothing which bears directly upon this subject. The suggestion is probably of an original character.

From *a priori* reasoning, that is, from argument based upon truths already in our possession, Dr. Svanberg's conclusions are not at all improbable.

No one can deny the importance of the subject.

For these reasons, we desire to call the attention of the profession to Dr. Svanberg's method. Certainly the external use of chloroform in labor is worthy of more extensive trial and more accurate investigation.

Dr. Svanberg's method of the external use of chloroform in labor is simple.

A piece of flannel, saturated with a solution of chloroform and sweet oil, equal parts, is applied to the skin, between the umbilicus and symphysis. Light strokes over the cloth secure even distribution of the solution, and exact approximation of the fabric. The application may be renewed according to necessity. Dr. Svanberg has observed that usually, in from five to ten minutes, the chloroform has performed its function.

The indications for the external use of chloroform in labor include a variety of conditions. In general terms, it may be said that the local application is designed to supersede the inhalation of chloroform.

Dr. Svanberg has successfully employed this method in cases of retained placenta with *tetanus uteri*, transverse presentations with rigidly contracted uterus and escape of the *liquor amnii*, and breech presentations with rigidity of the internal os. In certain of these cases, the inhalation of chloroform was not sufficient to relax the uterine contractions.

It is not unfitting, in this connection, to hear Dr. Svanberg's individual testimony as to the efficiency of the method: "Since that day, (December, 1877,) I have never used chloroform by inhalation for rigid contractions of the uterus."

